# Validation and Characterization of Platelet-Rich Plasma in the Feline: A Prospective Analysis

**DOI:** 10.3389/fvets.2020.00512

**Published:** 2020-08-11

**Authors:** Nicole Chun, Sherman Canapp, Brittany Jean Carr, Valerie Wong, Jeff Curry

**Affiliations:** ^1^Veterinary Orthopedic and Sports Medicine, Annapolis Junction, MD, United States; ^2^Orthobiologic Innovations, LLC, Annapolis Junction, MD, United States; ^3^IDEXX BioResearch, West Sacramento, CA, United States

**Keywords:** platelet-rich plasma, leukocyte poor platelet rich plasma, leukocyte rich platelet rich plasma, feline, platelet concentration

## Abstract

**Objective:** To quantitate key parameters of the platelet-rich plasma (PRP) product from a commercially available system^1^ in healthy, adult felines.

**Materials and methods:** A prospective study was performed from January 2019 to April 2019. 11 adult, healthy cats were used to prospectively analyze a commercially available PRP system^1^. A whole blood sample and a PRP sample that was processed immediately following blood draw according to the manufacturer's protocol were collected from each cat. The mean whole blood and PRP product platelet, RBC, WBC, neutrophil, monocyte, and lymphocyte concentrations were determined. The mean PRP product values were compared to the mean whole blood baseline values using a paired *t*-test with significance established at *p* = 0.05.

**Results:** Mean platelet concentration was significantly increased (*p* = 0.0155). Mean RBC concentration was significantly decreased (*p* < 0.0001). Mean neutrophil concentration was significantly decreased (*p* < 0.0001). There was no statistically significant difference in mean WBC, monocyte, and lymphocyte concentrations.

**Clinical Relevance:** The analyzed PRP system increased platelet concentration, while significantly reducing the RBC and neutrophil concentrations. Further study is warranted to determine the clinical applications and efficacy of PRP in felines, and the ideal concentrations of and relationships between platelets, red blood cells, and leukocytes needed for therapeutic effect.

## Introduction

Platelet-rich plasma (PRP) is an autologous blood concentrate of platelets. Platelets contain alpha granules that release growth factors to facilitate in tissue healing, including transforming growth factor-β1 and β2 (TGF-β1 and TGF-β2), platelet-derived growth factor (PDGF), vascular endothelial growth factor (VEGF), basic fibroblastic growth factor (bFGF), and epidermal growth factor (EGF) (1–6). Growth factors help with cellular migration, mitigation of inflammation, angiogenesis, and matrix deposition, making PRP beneficial for the treatment of osteoarthritis and soft tissue injuries (3, 5–7). ^1^ Numerous randomized controlled studies have looked at the efficacy of PRP for human knee osteoarthritis, showing that PRP can superiorly improve pain and function for up to 1 year when compared to other intra-articular therapies, including placebo, HA, saline and corticosteroids (7–10). Similar research in the canine is still in its infancy, but there are a growing number of reports demonstrating PRP's ability to alter the joint environment and improve clinical function in dogs with OA. Many of these studies involve the combined use of PRP with stem cells, as platelets have been shown to recruit and differentiate stem cells, enhance their survival, and provide them with scaffolding (11–16). Research has also been done on PRP for soft tissue injuries. In people, single injections of PRP for tendinopathies do not consistently improve pain scores, thickness, or Doppler activity (17–19). Rather, research shows better long-term results with a series of PRP injections, even in comparison to extracorporeal shockwave therapy (ESWT) and eccentric exercise (20).

Orthopedic disease in felines, particularly osteoarthritis, often went undiagnosed due to the subtly of clinical signs and potentially challenging physical examinations. Fortunately, there has been increased recognition of this disease in recent years, but this has led to new challenges regarding treatment options. Osteoarthritis more commonly affects older cats, a population that is also more likely to be afflicted with chronic kidney disease. Although studies have shown that meloxicam and robenacoxib are efficacious for treating arthritic pain in cats without increasing the risk of nephrotoxicity nor reducing survival time in those with CKD (21–26), many veterinarians are still reluctant to use NSAIDs long-term in these patients. Additionally, chronic NSAID use entails routine blood, urine, and blood pressure monitoring throughout treatment. Adjunctive drug therapy has been introduced to feline pain management regimens, but research is limited with variable results. In a recent study on gabapentin for the treatment of feline osteoarthritis, there was owner-assessed improvement in impaired activities, but overall activity counts were lower and sedation was commonly reported (27). Tramadol improved the activity levels of arthritic cats in one study (28), but in another had no added benefit when combined with meloxicam vs. meloxicam alone (29). Dietary supplementation with glucosamine and chondroitin could help slow cartilage degradation, but its analgesic potential and ability to improve mobility scores were lacking in comparison to meloxicam in a recent study (30).

The potential application of PRP in feline osteoarthritis is promising, but to date, there have been no prospective studies evaluating if PRP can be created from feline blood, what the optimal and desired cellular composition is for therapeutic effect, nor if such concentrations can be obtained. In humans, the reported ideal concentration of platelets is a 4–7-fold increase from baseline (3, 6). The concentration of red blood cells, neutrophils and mononuclear cells are also important components of the PRP product as they affect its efficacy and the inflammatory process (4, 31–33). PRP preparations can be leukocyte-rich (LR-PRP) or leukocyte-poor (LP-PRP or Pure PRP), in which leukocyte concentrations are above or below baseline concentrations, respectively. Generally, it is believed that red blood cells and leukocytes, particularly neutrophils, should be decreased because they are pro-inflammatory, delivering catabolic cytokines like interleukin-1β (IL-1β) and tumor necrosis factor-α (TNF-α) that induce cartilage degeneration in OA (4, 5, 31–34). The ideal concentration of monocytes and lymphocytes is still debatable, but there is increasing evidence that their increased concentration can be beneficial in tissue turnover and collagen synthesis, a potentially desirable effect for tendon and ligament injury (35–38).

Many PRP systems have been developed for use in humans, equine, and canines. Previous studies have shown that there are variations in platelet, leukocyte, and red blood cell concentrations between systems for a given species (39–42). Additionally, it has been shown that systems validated for humans and equines may not yield the same product parameters for canines (43), underlining the fact that the PRP product of one species may not necessarily be representative of the PRP product from another species using the same system. With each component of PRP potentially having a significant effect on the product's efficacy, one cannot stress enough the importance of characterizing the PRP product generated by a given system for the species to be treated. To the author's knowledge, no studies have been done looking at feline PRP products within any of the commercially available systems. Hence, the purpose of this study was to quantitate key parameters of the PRP product from a commercially available PRP system^1^ in healthy, adult felines.

## Materials and Methods

This is a prospective, validation study involving a single small animal surgery specialty center from January 2019 to April 2019. In accordance with AAALAC International Rules of Accreditation, this study was performed with the approval of the Veterinary Orthopedic and Sports Medicine Group Research Committee and with owner consent. All cats that participated in the study were client-owned and deemed healthy by a veterinarian. All clients volunteered their cat for the study and provided written consent as required by VOSM. All cats that participated in the study were directly overseen by a veterinarian to ensure no harm was incurred during study participation.

Eleven adult, healthy cats with no known previous or current medical problems were used to prospectively analyze a commercially available PRP system^1^. For each cat, a whole blood and PRP sample were collected in a single setting. Sample collections occurred between January and April 2019. Using a 22-gauge butterfly needle, 1.3 ml of blood was collected into a 1.3 ml EDTA tube for whole blood processing, and 12.5 ml of blood was collected into a 30 ml syringe containing 2.5 ml of Anticoagulant Citrate Dextrose Solution (ACDA) for processing in the concentrating device according to the manufacturer's protocol. Briefly, the 12.5 ml of whole blood was injected into the 30 ml concentrating device, placed into the centrifuge with a counterbalance, and spun for 1 min at 3,600 RPM. After centrifugation, a 30 ml syringe was attached to the concentrating device and the platelet-plasma suspension was aspirated from the device until RBC were seen in the top of the line. The platelet-plasma suspension was injected into another 30 ml concentrating device, placed into the centrifuge with a counterbalance, and spun for 5 min at 3,800 RPM. After centrifugation, a 30 ml syringe was attached to the concentrating device and all but 1 ml of plasma was aspirated from the device. The platelets were resuspended into the remaining plasma by gently swirling the device. A 12 ml syringe was attached to the device and the PRP was aspirated and placed in a 3 ml non-additive tube. The whole blood and PRP samples were immediately shipped overnight to IDEXX BioResearch. Upon receipt, WBC differential, RBC concentration, and platelet concentration were determined for all samples using a hematology analyzer that had been calibrated according to manufacturer standards. Platelet concentrations for all samples were corroborated by blood smears evaluated by one IDEXX BioAnalytics clinical pathologist.

The D'Agostino and Pearson Omnibus normality test was performed on the differences between PRP and whole blood for each data set with significance established at *p* = 0.01. All data sets were found to be normally distributed. The data was then analyzed using a paired *t*-test with significance established at *p* = 0.05. These values were analyzed using statistical software^2^.

## Results

Blood was collected from a total of 11 healthy adult felines. There were 5 spayed females and 6 neutered males. The mean weight was 4.7 kg (range 3.6–5.9 kg) and the mean age was 7.2 years old (range 2–12 years old).

### Platelet Concentration

**Figure 1:**
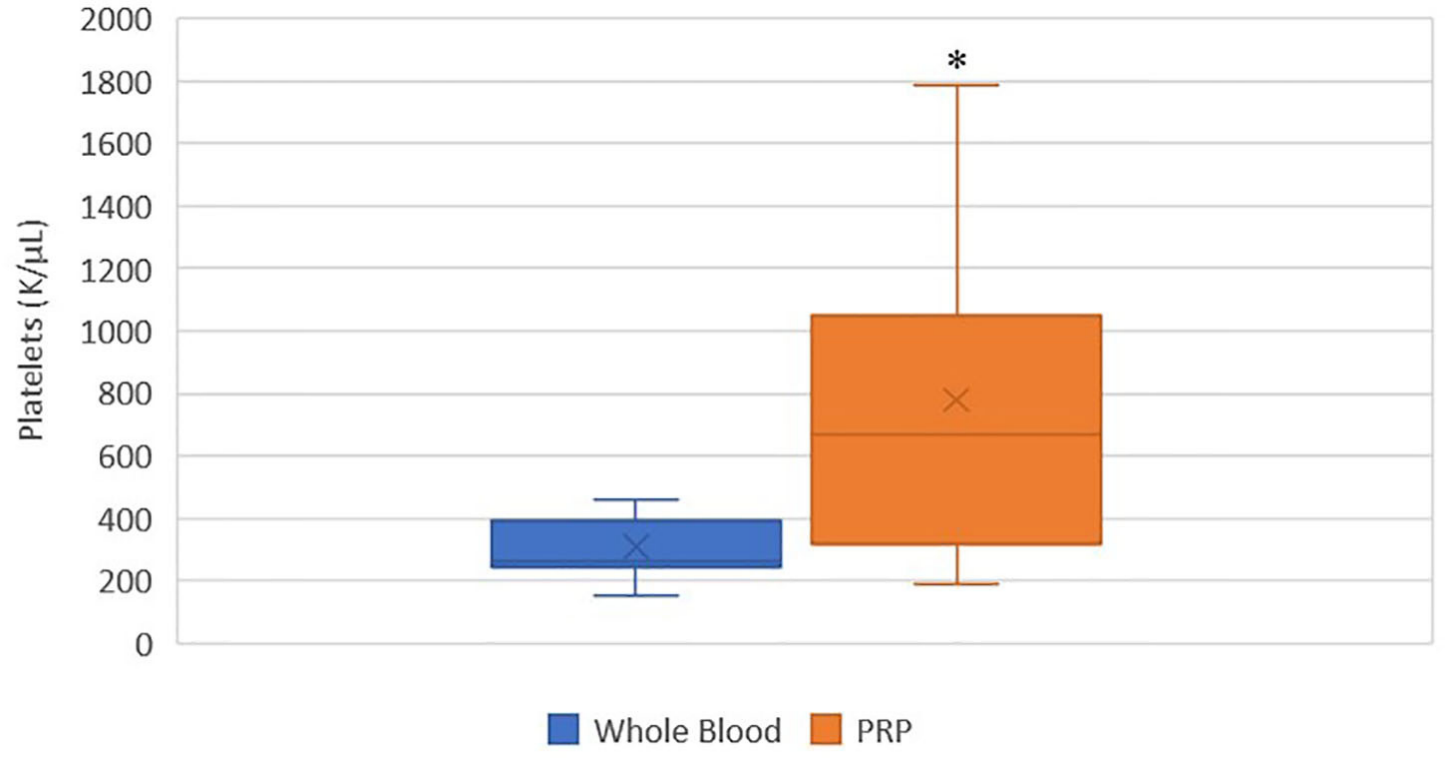
Comparison of mean whole blood and mean PRP product platelet concentrations. The middle line represents the median, the ends of the box are the 25th and 75th percentiles, the whiskers are the minimum and maximum values, and the “X” denotes the mean. *a statistically significant difference between the mean whole blood and mean PRP product platelet concentrations (*p* = 0.0155).

A statistically significant difference was found between the mean whole blood and mean PRP product platelet concentrations (Figure 1). Mean PRP product platelet concentration (777.7 K/μL ± 561.1) was significantly increased by 151% (*p* = 0.0155), a 2.5-fold increase in platelets from baseline on average. For all patients, microscopic evaluation of whole blood and PRP product smears confirmed enrichment of platelets in the PRP products. For cat 5, examination of the direct smear confirmed enrichment of platelets in the PRP product as compared to whole blood, but the analyzer was unable to detect this increase. Similarly, for cat 11, examination of the direct smear confirmed enrichment of the platelets in the PRP product as compared to whole blood, though minimal.

### RBC Concentration

**Figure 2:**
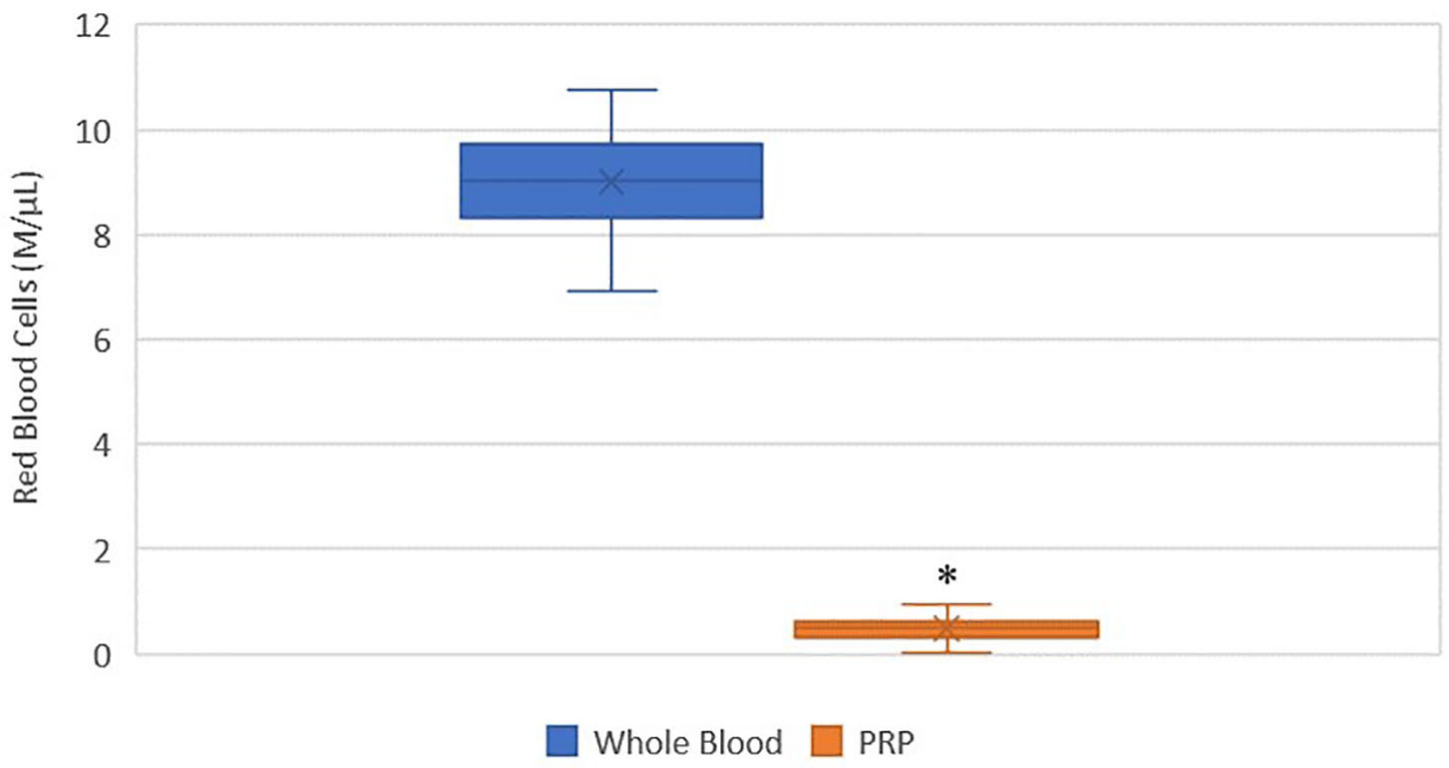
Comparison of mean whole blood and mean PRP product RBC concentrations. The middle line represents the median, the ends of the box are the 25th and 75th percentiles, the whiskers are the minimum and maximum values, and the “X” denotes the mean. *a statistically significant difference between the mean whole blood and mean PRP product RBC concentrations (*p* < 0.0001).

A statistically significant difference was found between the mean whole blood and mean PRP product RBC concentrations (Figure 2). Mean PRP product RBC concentration (0.5 M/μL ± 0.3) was significantly decreased by 95% (*p* < 0.0001), an 18.6-fold decrease in red blood cells from baseline on average. RBC concentration was decreased from baseline for all cats.

### WBC Concentration

**Figure 3:**
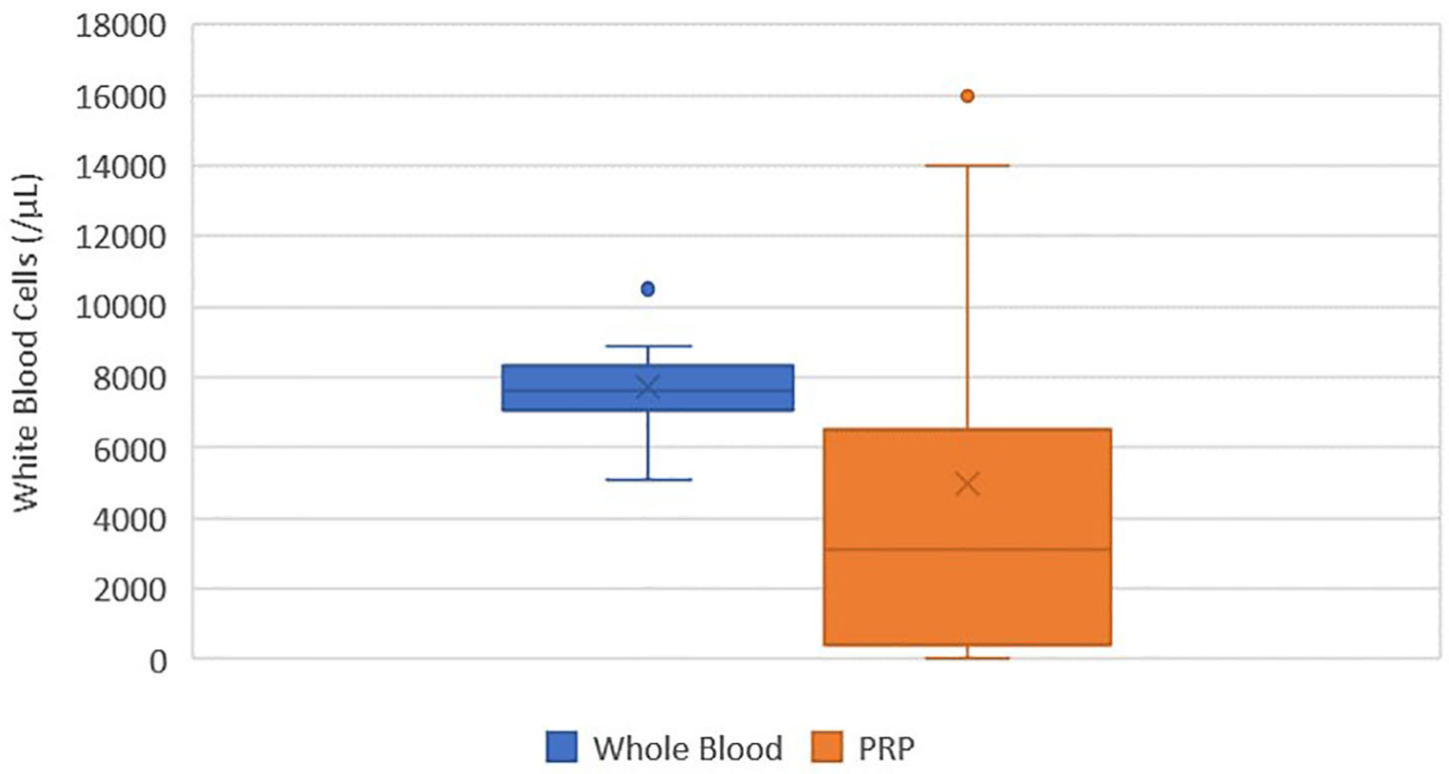
Comparison of mean whole blood and mean PRP product WBC concentrations. The middle line represents the median, the ends of the box are the 25th and 75th percentiles, the whiskers are the minimum and maximum values, and the “X” denotes the mean. There was no statistically significant difference between the mean whole blood and mean PRP product WBC concentrations (*p* = 0.1031).

There was no statistically significant difference between the mean whole blood and mean PRP product WBC concentrations (Figure 3). Mean PRP product WBC concentration (4,970/μL ± 5.6) was decreased by 36%, a 1.6-fold decrease in WBC from baseline on average, but was not statistically significant (*p* = 0.1031). WBC concentration was decreased from baseline for all cats, with exception of cat 1 (outlier) and cat 10, who experienced a 1.9-fold and a 1.6-fold increase in WBC concentration, respectively; these cats also had the smallest PLT:WBC ratios (Figure 7). PRP products of cats 2 and 7 had the lowest WBC concentrations, and had the largest PLT:WBC ratios. WBC were absent in the PRP product of cat 5, so a ratio could not be calculated.

### Neutrophil Concentration

**Figure 4:**
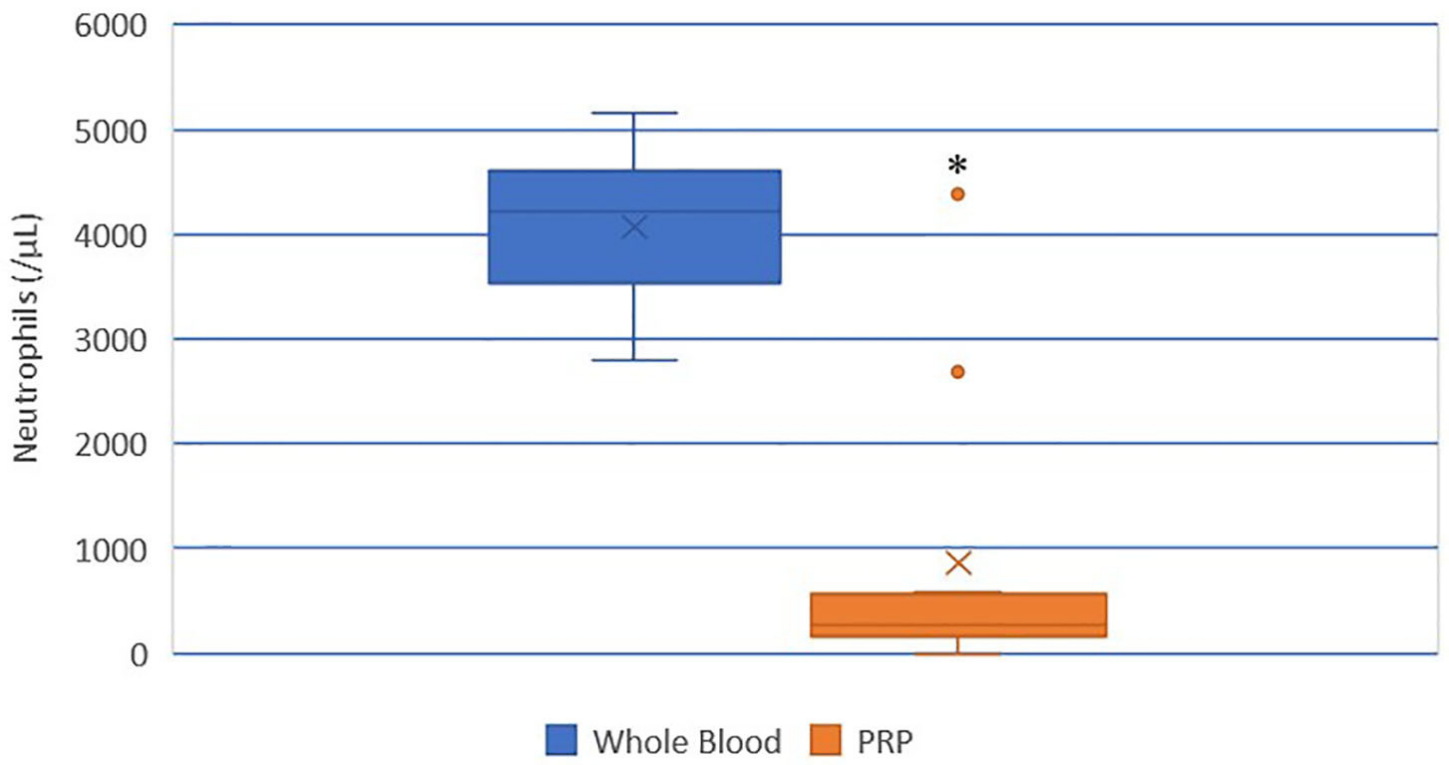
Comparison of mean whole blood and mean PRP product neutrophil concentrations. The middle line represents the median, the ends of the box are the 25th and 75th percentiles, the whiskers are the minimum and maximum values, and the “X” denotes the mean. *a statistically significant difference between the mean whole blood and mean PRP product neutrophil concentrations (*p* < 0.0001).

A statistically significant difference was found between the mean whole blood and mean PRP product neutrophil concentrations (Figure 4). Mean PRP product neutrophil concentration (857.7/μL ± 1,390) was significantly decreased by 79% (*p* < 0.0001), a 4.7-fold decrease in neutrophils from baseline on average. Neutrophil concentration was decreased from baseline in all cats, with the exception of cat 1 (outlier), who experienced no change in neutrophil concentration; this cat, in addition to cat 10 (outlier), had the smallest PLT:neutrophil ratios (Figure 7). Cat 2 had the lowest neutrophil concentration, and had the largest PLT:neutrophil ratio. Neutrophils were absent in the PRP product of cat 5, so a ratio could not be calculated.

### Monocyte Concentration

**Figure 5:**
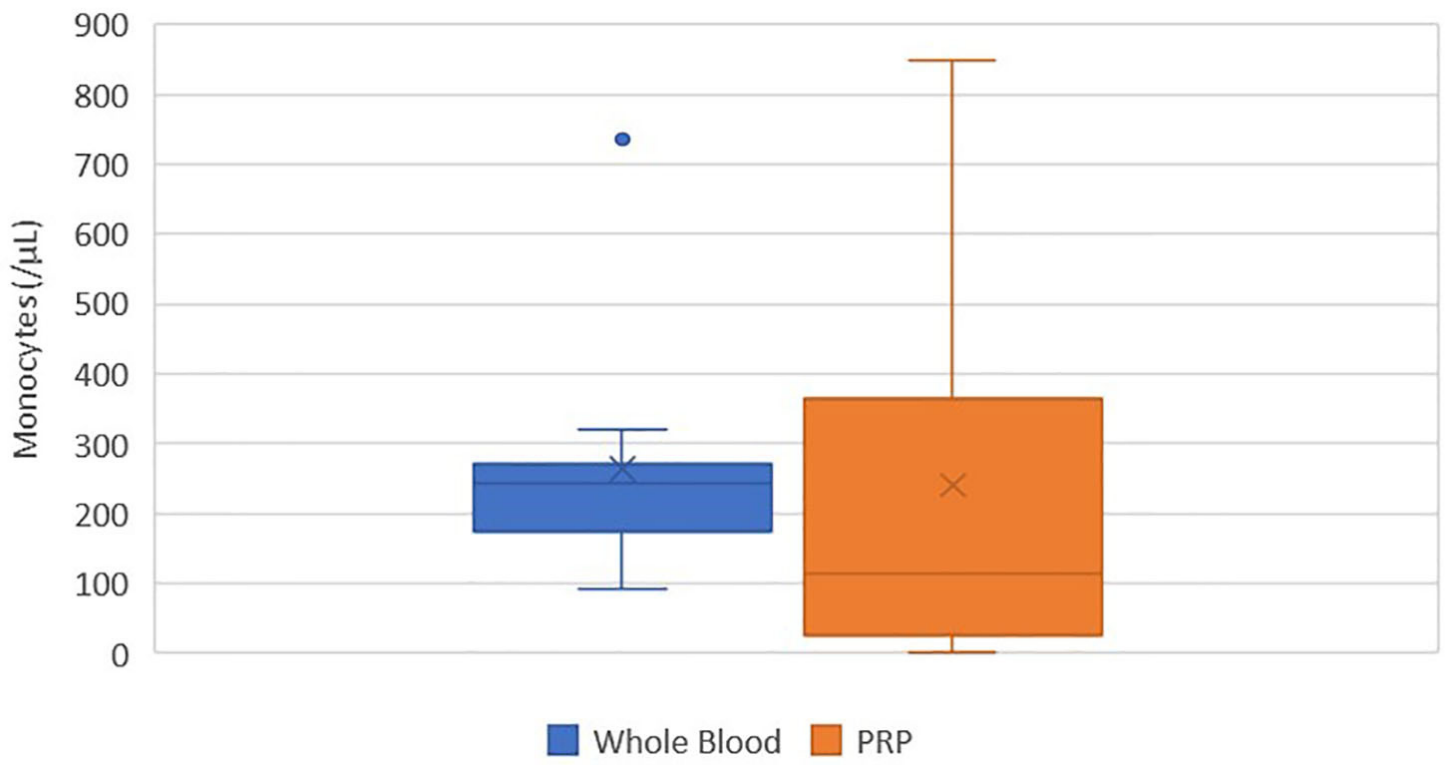
Comparison of mean whole blood and mean PRP product monocyte concentrations. The middle line represents the median, the ends of the box are the 25th and 75th percentiles, the whiskers are the minimum and maximum values, and the “X” denotes the mean. There was no statistically significant difference between the mean whole blood and mean PRP product monocyte concentrations (*p* = 0.8166).

There was no statistically significant difference between the mean whole blood and mean PRP product monocyte concentrations (Figure 5). Mean PRP product monocyte concentration (240.5/μL ± 284.1) was decreased by 8%, a 1.1-fold decrease in monocytes from baseline on average, but was not statistically significant (*p* = 0.8166). Monocyte concentration was decreased from baseline for all cats, with exception of cats 1, 3, 8, and 10, who experienced a 2.7, 1.7, 1.9, and 2.9-fold increase in monocyte concentration, respectively. Monocytes were absent in the PRP product of cat 5.

### Lymphocyte Concentration

**Figure 6:**
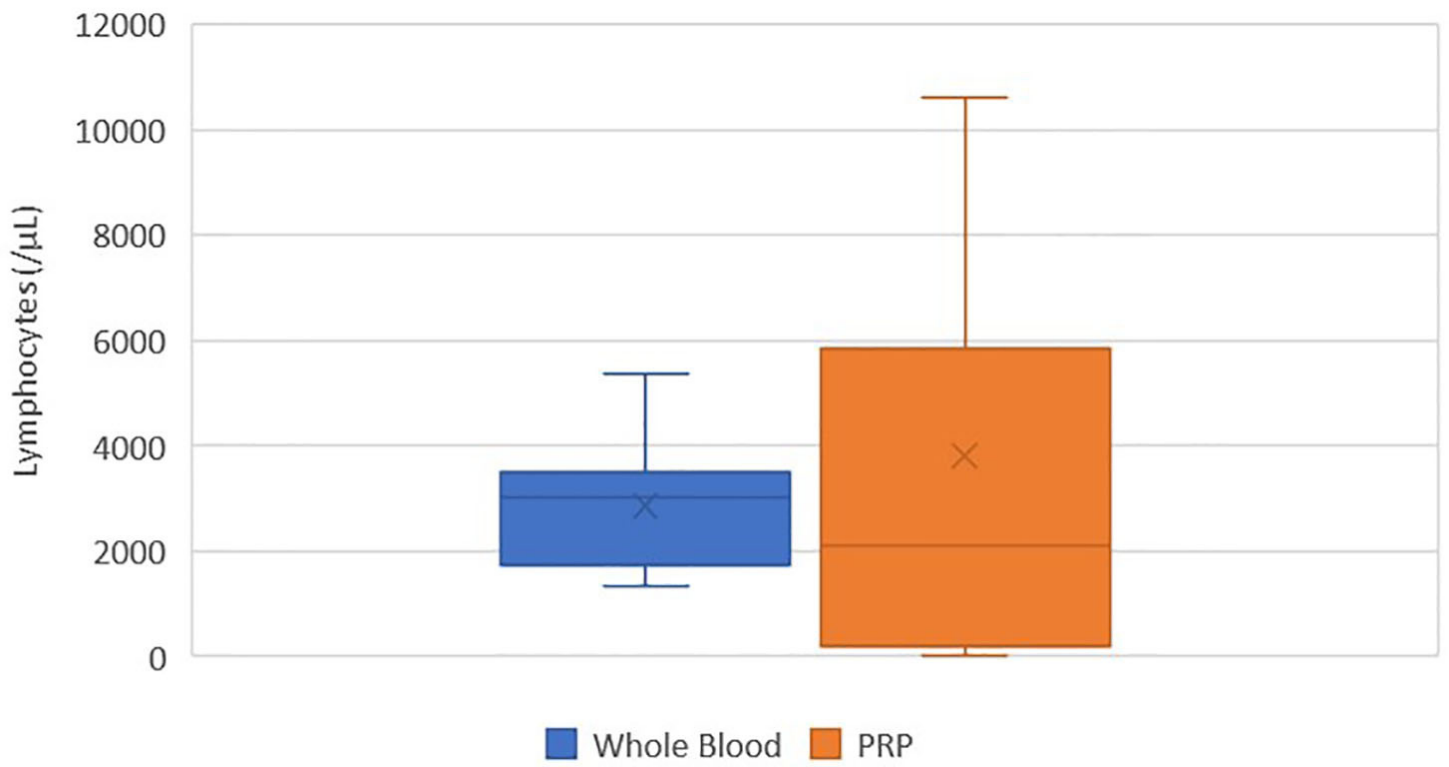
Comparison of mean whole blood and mean PRP product lymphocyte concentrations. The middle line represents the median, the ends of the box are the 25th and 75th percentiles, the whiskers are the minimum and maximum values, and the “X” denotes the mean. There was no statistically significant difference between the mean whole blood and mean PRP product lymphocyte concentrations (*p* = 0.3590).

There was no statistically significant difference between the mean whole blood and mean PRP product lymphocyte concentrations (Figure 6). Mean PRP product lymphocyte concentration (3805.4/μL ± 4010.8) was increased by 34%, a 1.3-fold increase in lymphocytes from baseline on average, but was not statistically significant (*p* = 0.3590). Lymphocyte concentration was increased from baseline for all cats, with exception of cats 2, 7, and 11, who experienced a 0.03, 0.03, and 1.6-fold decrease in lymphocyte concentration, respectively. Lymphocytes were absent in the PRP product of cat 5.

**Figure 7:**
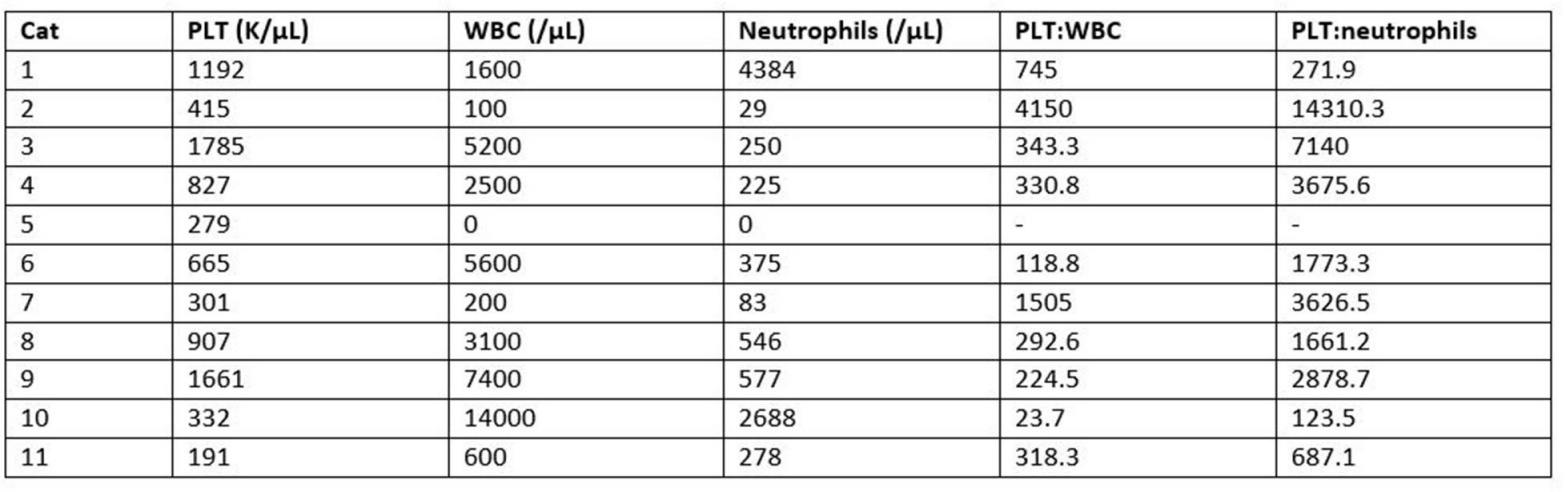
Comparison of individual PRP product WBC and neutrophil concentrations in relation to platelet concentration. Relationships are represented as ratios of PLT:WBC and PLT:neutrophils.

## Discussion

In this study, a commercially available PRP system^1^ was able to separate the key components in feline blood, significantly increasing mean platelet concentration by 151% (2.5-fold increase), while decreasing mean RBC and neutrophil concentrations by 95% (18.6-fold decrease) and 79% (4.7-fold decrease), respectively. However, this system did not significantly increase or decrease mean WBC, monocyte, nor lymphocyte concentrations compared to baseline.

Previous studies in humans report the ideal concentration of platelets in a PRP product should be 4–7-fold higher than baseline (3, 6). The commercial system analyzed in this study has been shown to increase platelets by 550% in canines (39). In the present study, the feline PRP product from this system had a 151% increase in platelet concentration, a 2.5-fold increase in platelets from baseline on average. Though lower than the reported ideal range, further investigation is required to determine whether this 2.5-fold increase in platelets is efficacious for varying orthopedic conditions in felines.

Platelets are not the only important component of PRP. PRP is also made up of red blood cells, neutrophils and mononuclear cells that affect the inflammatory process and efficacy of PRP (4, 31–33). Red blood cells are believed to be detrimental to the efficacy of the PRP product as they induce unwanted inflammatory mediators that can cause synoviocyte death and impede intra-articular healing (31). In this study, red blood cells were significantly reduced in all cats.

Leukocytes in PRP have also been previously studied, though the effect of their presence remains largely unknown. LR-PRP increases pro-inflammatory mediators and metalloproteinase gene expression, while downregulating cartilage oligomeric and decorin gene expression, effects that are largely caused by neutrophils (2, 31–33). Within an osteoarthritic joint, this can promote undesirable inflammation and result in synoviocyte death (4, 31, 33). In the current study, the PRP system did not significantly change the total WBC concentration, but did significantly reduce neutrophils. Similar findings were reported in previous study by Carr et al. in canines using this same system (39). Feline PRP products with a high PLT:neutrophil ratio would therefore be desirable, as seen in this study with cat 2. Interestingly, the platelets were not substantially increased compared to pre-spin amounts for that cat. Further research is indicated to determine whether a significant increase in platelets or a significant decrease in neutrophils are more critical to the efficacy of the PRP product.

Unlike osteoarthritis, tendinopathies may benefit from increased leukocyte concentrations, which is attributed to the presence of mononuclear cells. Mononuclear cells, particularly monocytes, have been associated with increased cellular metabolism and collagen production (35–38). In the study by Carr et al., lymphocytes and monocytes were increased using this system, but only lymphocytes reached significance (39). In this study, the PRP system increased lymphocytes and decreased monocytes on average, but neither reached significance. Cats 1 and 10 had increases in both lymphocytes and monocytes and had the smallest PLT:WBC ratios. However, these cats also did not substantially decrease neutrophils in comparison to the other cats. PRP products with smaller PLT:WBC ratios could be beneficial given the total increased leukocyte concentrations, but it is important to recognize which parameters are increasing the WBC concentration. Further study is warranted to determine the optimal concentrations of and relationships between neutrophils, monocytes, and lymphocytes in the final PRP product to achieve clinical efficacy for varying feline orthopedic conditions.

We must acknowledge that variation existed between the cats for each parameter, with the exception of RBC concentration, which was consistently decreased in all cats. The PRP products of cats 5 and 11 had decreased platelets. This may be a true decrease that reflects failure to create PRP in some cats, the incidence of which needs to be further elucidated with larger studies, or this may be a false decrease that may be attributed to either PRP processing error (failure to draw up entire platelet-plasma suspension) or failure of the hematology analyzer to detect the platelets. Regardless, this highlights the importance of analyzing all PRP product samples in both a hematology analyzer and under microscopy prior to application. There was variation in leukocytes between individual cats; not all cats had PRP product values that reflected the mean. The clinical significance of lymphocyte and monocyte variation between cats is unknown due to their indefinite role in PRP, but the failure to reduce neutrophils in cat 1 is concerning due to the potential of neutrophils to cause unwanted inflammation. The PRP product from cat 5 had no leukocytes, which is likely a processing error. In summary, the studied PRP system can reliably decrease red blood cells in cats, but a larger sample size is indicated to assess the true variability of leukocyte counts, which may affect the clinical efficacy of the final product.

One limitation of this study is the small sample size. As previously mentioned, a larger sample size is needed to determine true variability of PRP product parameters between cats. A larger sample size may have also the increased the average platelet concentration into the reported ideal range and produce significance in WBC, monocyte, and lymphocyte concentrations, but power analysis was not performed in this study.

This study aimed to quantitate key parameters of the PRP product from a commercially available system^1^ in healthy, adult felines. The parameters produced in this study cannot be assumed to be representative of a feline PRP product created by another PRP system. Additionally, no claims are made regarding the efficacy of the PRP formulations evaluated. Further study is needed to determine the ideal concentrations of platelets, red blood cells, and leukocytes needed for therapeutic effect. Additional research is also indicated to determine the clinical applications and efficacy of PRP in felines, and whether the ideal concentrations vary depending on the condition treated.

## Data Availability Statement

The datasets generated for this study are available on request to the corresponding author.

## Ethics Statement

The animal study was reviewed and approved by AAALAC. Written informed consent was obtained from the owners for the participation of their animals in this study.

## Author Contributions

All authors assisted with study development, procedures, data analysis, and manuscript preparation.

## Conflict of Interest

The authors declare that this study received funding from Companion Regenerative Therapies and Orthobiologic Innovations. The funders and SC were involved in the study design and the donation of regenerative medicine products and system, but were not involved in the collection, analysis, and interpretation of the data, nor the writing of this article and decision to submit it for publication. SC is a paid consultant for Companion Regenerative Therapies and an owner of Orthobiologic Innovations. JC is a paid researcher for Orthobiologic Innovations. The remaining authors declare that the research was conducted in the absence of any commercial or financial relationships that could be construed as a potential conflict of interest.

## Footnotes

^1^Companion Regenerative Therapies PurePRP system, Newark, DE.

^2^Microsoft Excel, Redmond, WA.
